# Long-Term Survival and Mortality in Schizophrenia: Insights from a 10-Year Follow-Up Study

**DOI:** 10.1192/j.eurpsy.2025.418

**Published:** 2025-08-26

**Authors:** A. V. Popa, A. Teodorescu, P. I. Ifteni

**Affiliations:** 1 Faculty of Medicine, Transilvania University of Brașov; 2 Psychiatry, Clinical Hospital of Psychiatry and Neurology of Brașov, Brașov, Romania

## Abstract

**Introduction:**

Schizophrenia is a severe mental disorder linked to a life expectancy 15-20 years shorter than the general population^1^, due to higher rates of cardiovascular disease, cancer, metabolic disorders, and increased risk of suicide and accidental deaths^2^.

**Objectives:**

This study aims to analyze survival and causes of death in a cohort of schizophrenia patients over a 10-year period, providing insights into mortality patterns in this population.

**Methods:**

This 10-year retrospective study followed 635 schizophrenia patients, aged 18 or older, enrolled from 2010 to 2013 at the Clinical Hospital of Psychiatry and Neurology, Brasov, Romania. Patients with schizo-affective or other psychotic disorders were excluded. Data included demographics, clinical history, and survival outcomes, with causes of death confirmed by a Forensic Medical Specialist.

**Results:**

The study included 635 patients diagnosed with schizophrenia. The mean age at baseline was 48,01 ± 11.36, 42.04% were males, and the mean age of onset of schizophrenia was 26.68 ± 8.01.The average duration of illness was 21.27 ± 11.41 years. Among the cohort, 20.31% patients were treated with LAIs antipsychotics, and 17.16% were on clozapine. Of the 635 patients followed, 123 (19.4%) died during the 10-year follow-up. The average age at death was 59.04±11.96. According to the 2023 Eurohealth report and the World Health Organization, the overall life expectancy in Romania is 76.3 years ^3^. The data on schizophrenia patients suggests a significant disparity between their average age at death and the overall life expectancy in Romania. Schizophrenia patients in Romania live, on average, about 17 years less than the general population. Of the deceased, 13% died in psychiatric wards, and 17.88% were in chronic care at the time. Among the deceased patients, 18 were on typical antipsychotic LAIs. None of the patients in the deceased cohort were on atypical LAIs. Cardiovascular disease was the leading cause of death (27.64%), followed by infections (17.07%) and cancer (12.19%). Metabolic causes accounted for 4.06%, respiratory for 1.62%, hepatic for 3.25%, and both neurological and gastrointestinal causes for 0.81%. The cause of death was undetermined in 15.45% of cases. Violent deaths accounted for 17.07% of cases, with 8 suicides and 13 accidents. Out of the 13 accidental deaths, 7 were due to choking-related asphyxiation during eating. Four of these patients were on haloperidol, 2 on quetiapine, and 1 on flupenthixol.

**Conclusions:**

The 17-year lower life expectancy for schizophrenia patients highlights the urgent need for targeted public health interventions and improved preventive care. Additionally, the high mortality from cardiovascular disease, cancer, and infections, along with choking-related risks from antipsychotic medications, underscores the importance of careful medication management to enhance patient safety and survival.

**Disclosure of Interest:**

None Declared

